# Stimulatory Effect of Intermittent Hypoxia on the Production of Corticosterone by Zona Fasciculata-Reticularis Cells in Rats

**DOI:** 10.1038/s41598-017-07054-6

**Published:** 2017-08-22

**Authors:** Guey-Shyang Hwang, Chih-Chieh Chen, Jou-Chun Chou, Ling-Ling Chang, Shu-Fen Kan, Wei-Ho Lai, Fu-Kong Lieu, Sindy Hu, Paulus S. Wang, Shyi-Wu Wang

**Affiliations:** 1 Department of Physiology, School of Medicine, National Yang-Ming University, Taipei, 11221 Taiwan; 2 Medical Center of Aging Research, China Medical University Hospital, Taichung, 40402 Taiwan; 3 Department of Life Sciences, National Chung Hsing University, Taichung, 40254 Taiwan; 4 Department of Chemical and Materials Engineering, Chinese Culture University, Taipei, 11114 Taiwan; 5 Department of Rehabilitation, Cheng Hsin General Hospital, Taipei, 11212 Taiwan; 6 Department of Medical Research, Taipei Veterans General Hospital, Taipei, 11217 Taiwan; 7 Graduate Institute of Basic Medical Science,College of Medicine, China Medical University, Taichung, 40402 Taiwan; 8 Department of Physiology and Pharmacology, College of Medicine, Chang Gung University, Taoyuan, 33302 Taiwan; 9 Department of Nursing, Chang Gung University of Science and Technology, Taoyuan, 33303 Taiwan; 10 Aesthetic Medical Center, Department of Dermatology, Chang Gung Memorial Hospital, Taoyuan, 33378 Taiwan; 11 Department of Biotechnology, College of Health Science, Asia University, Taichung, 41354 Taiwan Republic of China

## Abstract

Hypoxia or intermittent hypoxia (IH) have known to alter both synthesis and secretion of hormones. However, the effect of IH on the production of adrenal cortical steroid hormones is still unclear. The aim of present study was to explore the mechanism involved in the effect of IH on the production of corticosterone by rat ZFR cells. Male rats were exposed at 12% O_2_ and 88% N_2_ (8 hours per day) for 1, 2, or 4 days. The ZFR cells were incubated at 37 °C for 1 hour with or without ACTH, 8-Br-cAMP, calcium ion channel blockers, or steroidogenic precursors. The concentration of plasma corticosterone was increased time-dependently by administration of IH hypoxia. The basal levels of corticosterone production in cells were higher in the IH groups than in normoxic group. IH resulted in a time-dependent increase of corticosterone production in response to ACTH, 8-Br-cAMP, progesterone and deoxycorticosterone. The production of pregnenolone in response to 25-OH-C and that of progesterone in response to pregnenolone in ZFR cells were enhanced by 4-day IH. These results suggest that IH in rats increases the secretion of corticosterone via a mechanism at least in part associated with the activation of cAMP pathway and steroidogenic enzymes.

## Introduction

During the past few years, the investigation of altitude hypoxia and its effect on metabolic functions in humans has increasingly attracted the attention of endocrinologists. For example, the secretion of erythropoietin, a hormone which is well known to stimulate erythropoiesis can be increased by hypoxia^1^. Several endocrine parameters were shown to be affected by altitude hypoxia including erythropoietin, cortisol, thyroxine, triiodothyronine and interleukin 6 (IL-6)^2^. The hypophyseal hormones are also subjected to a hypoxia-induced decrease in their response to hypothalamic factors^3^. The cellular responses, e.g. metabolism, fetal immune system, and glucocorticoid signaling, to hypoxia were reduced^4^. It has been suggested that the stimulatory action of acute hypoxia on somatostatin (SS) content in rat median eminence (ME) is due to the increased corticosterone levels during hypoxia^5^.

Resistance exercise in hypoxic condition significantly increases the secretion of lactate, GH, cortisol, epinephrine, norepinephrine, insulin-like growth factor 1 and testosterone^6^. Furthermore, swimmers exposed at altitude hypoxia (2,300 m) showed improved skeletal muscle mass and 11.4% reduction of body mass after 3 weeks^7^. These results revealed that the exercise performance could be enhanced by the hypoxia training. High-altitude hypoxia induces disorders of the brain-endocrine-immune network through activation of corticotropin-releasing hormone (CRH) and corticotropin-releasing hormone receptor 1 (CRHR1) in the brain and periphery including activation of the hypothalamus–pituitary–adrenal (HPA) axis in a time- and dose-dependent manner^7^. Prospective studies have suggested an association between hypertension and obstructive sleep apnea syndrome (OSAS) because intermittent nocturnal hypoxia prompts an increase in sympathetic tone, endothelial dysfunction, and vascular inflammation: aldosterone excess may have a pathophysiological role^8^. Hypoxia induces dysfunctions of the immune systems, including suppression of growth and development, as well as inhibition of reproductive, metabolic and immune functions^9^.

Exposure to hypoxia for 7 days from birth resulted in a marked increase in rat plasma ACTH, corticosterone, and aldosterone without change of plasma renin activity (PRA)^10^. Recent studies demonstrated that hypoxia may upregulate specific steroidogenic enzymes and hormone receptors through actions of miRNA, and hence provide a novel mechanism for the observed female reproductive impairment caused by hypoxia^11^. The hypoxia/ischemia (HI) induced impairment in synaptic transmission in the hippocampal CA1 area *in vitro* was exacerbated by concomitant corticosteroid treatment and alleviated by pretreatment with metyrapone^12^. It has been shown that the synaptic functions along with cellular integrity are preserved after preventing the ischemia-evoked rise in corticosteroid levels rather than blocking the glucocorticoid receptor^12^.

It is well known that stress is a complex condition associated with emotional, cognitive, and biological factors. Excessive stress causes long- and short-term disability in the various human systems, and activates the defense system of the central nervous system. CRH launched a wave of discoveries that resulted in the significant achievement of what we know about stress physiology and stress-related pathologies^13^. Stressful events also activate the HPA axis. This results in the release of CRH which stimulates the release of ACTH from the pituitary gland and finally the release of glucocorticoid hormones from the adrenal cortex (corticosterone in most rodents; cortisol in humans). Adrenal corticosteroid synthesis and its regulation have been reviewed extensively elsewhere^14^. Corticosterone is synthesized in the zona fasciculata of the adrenal cortex through the actions of four enzymes named cholesterol desmolase, 3β-hydroxysteroid dehydrogenase (3β-HSD), 21β-hydroxylase and 11β-hydroxylase, which are cytochromes P450 (CYP), membrane-bound heme-containing enzymes that accept electrons from nicotinamide adenine dinucleotide phosphate H (NADPH) via accessory proteins and utilize molecular oxygen to perform hydroxylations or other oxidative conversions^15^.

Since hypoxia has been known as a stress, it is reasonable to expect a changed response of stress such as ACTH and glucorticoid^16^. It has been shown that the rise of intracellular Ca^2+^ in response to stress has been mainly attributed to the opening of voltage gated L-type Ca^2+^ channels^17^. Stress-induced changes in Ca^2+^ currents have been mainly attributed to increased release of corticosterone and catecholamine^17, 18^. However, the interaction between hypoxia and corticosterone release as well as the involvement of signalling pathway and steroidogenic enzymes are not clear. In the present study, our hypothesis is that intermittent hypoxia acts through the pathways of the actions of ACTH and cAMP, calcium channel, and cholesterol side-chain cleavage enzyme (P450scc) to affect the secretion of corticosterone by zona fasciculata-reticularis cells. The objectives of the present work are to explore the effects of intermittent hypoxia (8 hours per day for 0, 1 or 4 days) on the release of corticosterone in response to some stimulants including ACTH, 8-Br-cAMP, Ca^2+^ channel blockers, and steroidogenic precusors to detect the involvement of cAMP pathway, calcium channels and activities of steroidogenic enzymes during biosynthesis and secretion of corticosterone.

## Materials and Methods

### Materials

Bovine serum albumin (BSA), N-2-hydroxy-ethylpiperazine-N’-2-ethane-sulphonic acid (HEPES), Hank’s balanced salt solution (HBSS), glucose, collagenase, nifedipine, ACTH, 8-Br-cyclic AMP, 25-hydroxy-cholesterol, and deoxycorticosterone were purchased from Sigma Chemical Co. (St. Louis, MO, USA). Nimodipine was purchased from RBI/Sigma (Natick, MA, USA). Tetrandrine was purchased from Sigma-Aldrich. Trilostane was a gift from Sanofi-Synthelabo, Inc. (Malvern, PA, USA). [^3^H]-corticosterone, [^3^H]-pregnenolone and [^3^H]-progesterone were obtained from Amersham International Plc. (Bucks, UK). MC2-R (ACTH receptor, sc-13107) was purchased from Santa Cruz (Santa Cruz, CA, USA). Cytochrome P450 side-chain-cleavage enzyme (P450scc, bs-3608R) was purchased from Bioss Inc. (Woburn, MA, USA). The anti-steroidogenic acute regulatory protein (StAR) as a gift and kindly provided by Dr. Douglas Stocco, Department of Cell Biology and Biochemistry, Texas Tech University Health Sciences Center, Lubbock, TX, USA.

### Animals

Male Sprague-Dawley rats weighing 300–350 g were provided from Animal Center of National Yang-Ming University and housed in a temperature-controlled room (22 ± 1 °C) with 14 h of artificial illumination daily (0600–2000). Food and water were given *ad libitum*. The use of the animals was approved (No. 2014019) by the Institutional Animal Care and Use Committee of the Chang Gung University of Science and Technology. All animals received human care in compliance with the Principles of Laboratory Animal Care and the Guide for the Care and Use of Laboratory Animals published by the National Science Council, Taiwan. The rats were randomly assembled to form 3 groups: [1] Exposed to normoxia condition (control group); [2] Exposed to intermittent hypoxia (12% O_2_; 88% N_2_, 8 hours per day) for 1 day; and [3] Exposed to intermittent hypoxia (12% O_2_; 88% N_2_, 8 hours per day) for 4 days. Intermittent hypoxia was performed from 08:30 AM to 16:30 PM daily (8 hours per day) in a plastic chamber (L × W × H = 24.5 cm × 19 cm × 32 cm) containing 50–65 g sodasorb (W. R. Grace S. A., Epernon Cedex, France) for absorbing CO_2_. Since hypoxia has been also known as a stress, thus the continuous hypoxia might be harmful or more damaging to the animal as compared with intermittent hypoxia, in which animals may be recovered or restored from a stress, i.e. hypoxia condition. This also means that animals or tissues in the body returned to the physiological condition after a stress. After the last administration of intermittent hypoxia, rats were allowed to rest for 16 h and sacrificed on the next day.

### Effects of hypoxia on the plasma concentration of corticosterone in male rats

The normoxic and hypoxic rats were decapitated and the trunk blood samples were collected. The plasma was separated by centrifugation at 1000 × g for 30 min at 4° C. To measure the level of plasma corticosterone, the plasma was mixed with diethyl ether (Merck, Darmstadt, Germany) and shaken for 30 min. Then, quickly froze in the mixture of acetone and dry ice. The organic phase was collected and dried. The samples were reconstituted with assay buffer before the corticosterone RIA.

### Preparation of zona fasciculata-reticularis (ZFR) cells

An adrenocortex preparation enriched with zona fasciculata-reticularis cells for culture was performed following a method described elsewhere by Purdy *et al*.^19^ with minor modification^20, 21^. After decapitation, rat adrenal glands were rapidly excised and stored in ice-cold 0.9% NaCl solution. The encapsulated glands were separated into outer zone (mainly zona glumerulosa) and inner zone (mainly zona fasciculata and zona reticularis) fractions (ZFR) with forceps. The fraction of inner zone from 6–8 adrenals from different rats was incubated with collagenase (2 mg/ml) at 37 °C in a vibrating waterbath, 100–110 strokes per min, for 60 min. The collagenase was dissolved in 2–4 ml of Krebs-Ringer bicarbonate buffer (3.6 mM K^+^, 11.1 mM glucose) with 0.2% bovine serum albumin (KRBGA), pH 7.4. The ZFR cells were dispersed by repeated pipetting and filtered through a nylon mesh. After centrifugation at 200 × g for 10 min, the cells were washed in KRBGA medium and centrifuged again. Erythrocytes were separated by hypotonic shock with 9 ml deionized water for a few seconds. The ZFR cells were then mixed with 1 ml of 10 × Hank’s balanced salt solution (HBSS, pH 7.4). After centrifugation at 200 × g for 10 min, the supernatant was discarded and the pellets were resuspended in 3 ml of KRBGA medium. An aliquot (20 μl) was used to count the cells in a hemocytometer after staining with 0.05% nigrasin stain. The viability of isolated ZFR cells was 70–75%. Cells in the culture medium were further diluted to a concentration of 5 × 10^4^ cells/ml and the suspension was divided into the test tubes.

### Effects of hypoxia on the basal, ACTH- or 8-Br-cAMP-stimulated corticosterone release and the activity of calcium channel in ZFR cells

To measure the effects of hypoxia on basal and cyclic AMP-related corticosterone release, the ZFR cells (5 × 10^4^ cells/ml) were preincubated at 37° C under 95% O_2_–5% CO_2_ for 60 min prior to incubation with 0.5 ml medium containing ACTH (10^−9^ M), or 8-Br-cAMP (a membrane permeable analogue of cyclic AMP, 10^−5^ M) for 60 min. At the end of the incubation, the incubation tubes were centrifuged at 100 × g for 10 min at 4° C. The supernatant was collected for measurement of corticosterone by RIA. To identify the involvement of different types of Ca^2+^ channels in the hypoxia-related effects of corticosterone release, nifedipine, nimodipine (L-type calcium channel blockers), and tetrandrine (a blocker for T-type calcium channels) were employed. The isolated ZFR cells (5 × 10^4^ cells) were exposed with Ca^2+^ channel blockers, including nifedipine (10^−5^ M), nimodipine (10^−5^ M), and tetrandrine (10^−5^ M) for 60 min. At the end of incubation, the media were collected for RIA.

### Effects of hypoxia on the steroidogenesis of corticosterone in ZFR cells

Several enzymes involved in the biosynthesis of corticosterone in ZFR cells were examined. For studying the influence of hypoxia on the early step of enzyme activity of steroidogenesis, rat ZFR cells were incubated with 0.5 ml KRBGA medium containing precursors of steroidogenesis such as [1]25-OH-cholesterol (a membrane permeable cholesterol, 10^−5^ M) plus trilostane (an inhibitor of 3β-HSD, 10^−6^ ~ 10^−5^ M), and [2] pregnenolone (the substrate of 3β-HSD, 10^−6^ M) for l h. The media were collected and the concentrations of pregnenolone and progesterone in media were measured by RIA.

### Corticosterone RIA

The concentrations of corticosterone in both plasma and medium were determined by RIA as described elsewhere^21^. With anti-corticosterone No. PSW# 4–9, the sensitivity of corticosterone was 5 pg per assay tube. The intra- and interassay coefficients of variation were 3.3% (n = 5) and 9.5% (n = 4), respectively.

### Pregnenolone RIA

The concentrations of pregnenolone were measured by RIA developed in our laboratory^22^. The cross-reactivities of the anti-pregnenolone antibody were 67% with pregnen-36-OL-20-ONE sulphate; 19% with progesterone; and <3% with 17α-hydroxypregnenolone, cholesterol, 17α-OH-progesterone, 20α-diOH-progesterone, cortisol, deoxycorticosterone, corticosterone, aldosterone, androstenedione, testosterone, estradiol, estrone, or estriol. For the RIA system, a known amount of unlabelled pregnenolone or an aliquot of rat ZFR cell medium, adjusted to a total volume of 0.3 ml with a buffer solution [0.1% gelatin-phosphate-buffered saline (PBS), pH 7.5], was incubated with 0.1 ml pregnenolone antiserum diluted with 0.1% gelatin-PBS plus 0.1 ml [^3^H]pregnenolone at 4 °C for 24 h. Duplicate standard curves for pregnenolone were incubated in each assay. An adequate amount (0.1 ml) of dextran-coated charcoal (0.5%) was added and further incubated in an ice bath for 15 min. After incubation, the assay tubes were centrifuged at 1500 × g for 40 min. The supernatant was mixed with 3 ml liquid scintillation fluid (Ready Safe) before the radioactivity was counted in an automatic beta counter (Wallac 1409, Pharmacia, Turku, Finland). The sensitivity of the pregnenolone RIA was 16 pg per assay tube. The inhibition curves produced by ZFR cell medium samples were parallel to those produced by pregnenolone. The intra- and interassay coefficients of variation were 2.5% (n = 4) and 3.9% (n = 5), respectively.

### Western blot

The ZFR cells were purified from adrenal glands. Purified cells were lysed and homogenized by the RIPA buffer (50 mM Tris-HCl, pH 7.4, 1% NP-40, 1 mM EDTA, 10 μg/ml leupeptin, 10 μg/ml pepstatin A, 5 μg/ml NaF, 5 μg/ml aprotinin, 1 mM PMSF). After centrifugation, the supernatant was collected and quantified protein content by the Bradford reagent (Bio-Rad, Hercules, CA, USA). The same concentration of 40 μg/ml protein loaded into the 10% SDS-PAGE and transferred to the PVDF membrane (Millipore, Billerica, MA, USA). The primary antibody against ACTH receptor (ACTH-R), StAR and P450scc, and β-actin was used as a loading control. The horseradish peroxidase-conjugated rabbit or mouse secondary antibodies were employed with a chemiluminescent detection system as described by an instrument (LAS-4000 mini, Fujifilm, Tokyo, Japan).

### Progesterone RIA

The concentrations of progesterone in the media were determined by RIA as described elsewhere^23^ with anti-progesterone serum No. W5, the sensitivity of progesterone RIA was 5 pg per assay tube. Intra- and interassay coefficients of variation (CV) were 4.8% (n = 5) and 9.5% (n = 4), respectively.

### Statistical analysis

All values are given as the means ±S.E.M. In some cases, the treatment means were tested for homogeneity by two-way analysis of variance (ANOVA), and the difference between specific means was tested for significance by Dunnett’s test and Duncan’s multiple-range test^24^. To examine the difference between two means, Student’s *t*-test was employed. The relationship between treatments including the intermittent hypoxia and protein expressions of ACTH-R were analyzed by Pearson’s correlation analysis (IBM SPSS statistics 22). A difference between two means was considered statistically significant when *P* < 0.05.

## Results

### Effects of intermittent hypoxia on the plasma levels of corticosterone in male rats

**Figure 1:**
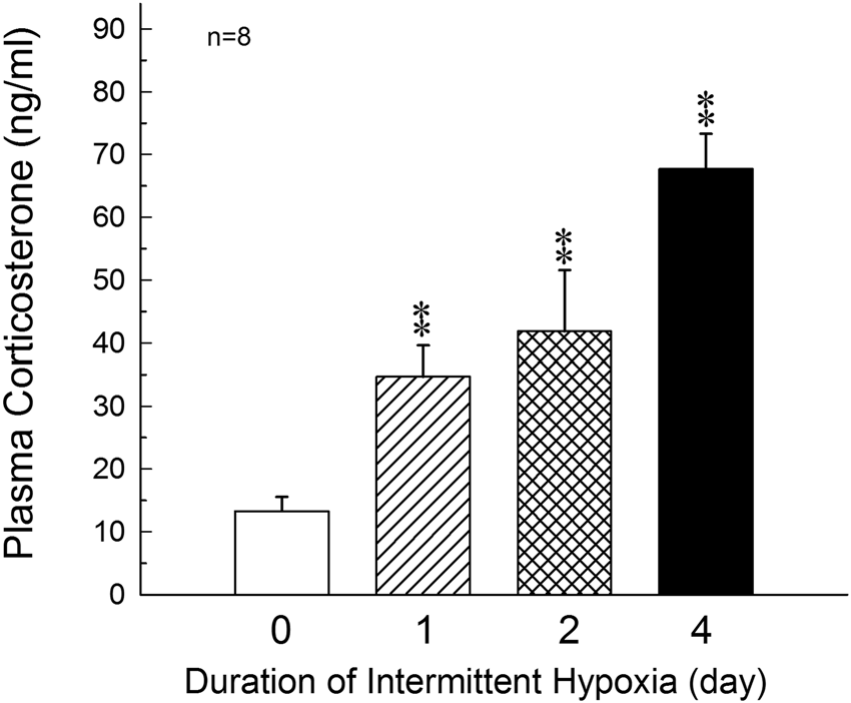
Effects of hypoxia on the plasma levels of corticosterone in male rats. *P < 0.05, **P < 0.01 compared to normoxia (control) group. Each value represents the mean ± SEM.

The change of plasma corticosterone concentration during the exposure of intermittent hypoxia was first accessed. Compared with normoxia (control) group, the concentrations of corticosterone were significantly elevated after treatment of intermittent hypoxia for 1 to 4 days (F = 38.72, *P* < 0.01, Fig. 1).

### Effects of intermittent hypoxia *in vivo* on the basal, ACTH- or 8-Br-cAMP-stimulated corticosterone release in ZFR cells

**Figure 2:**
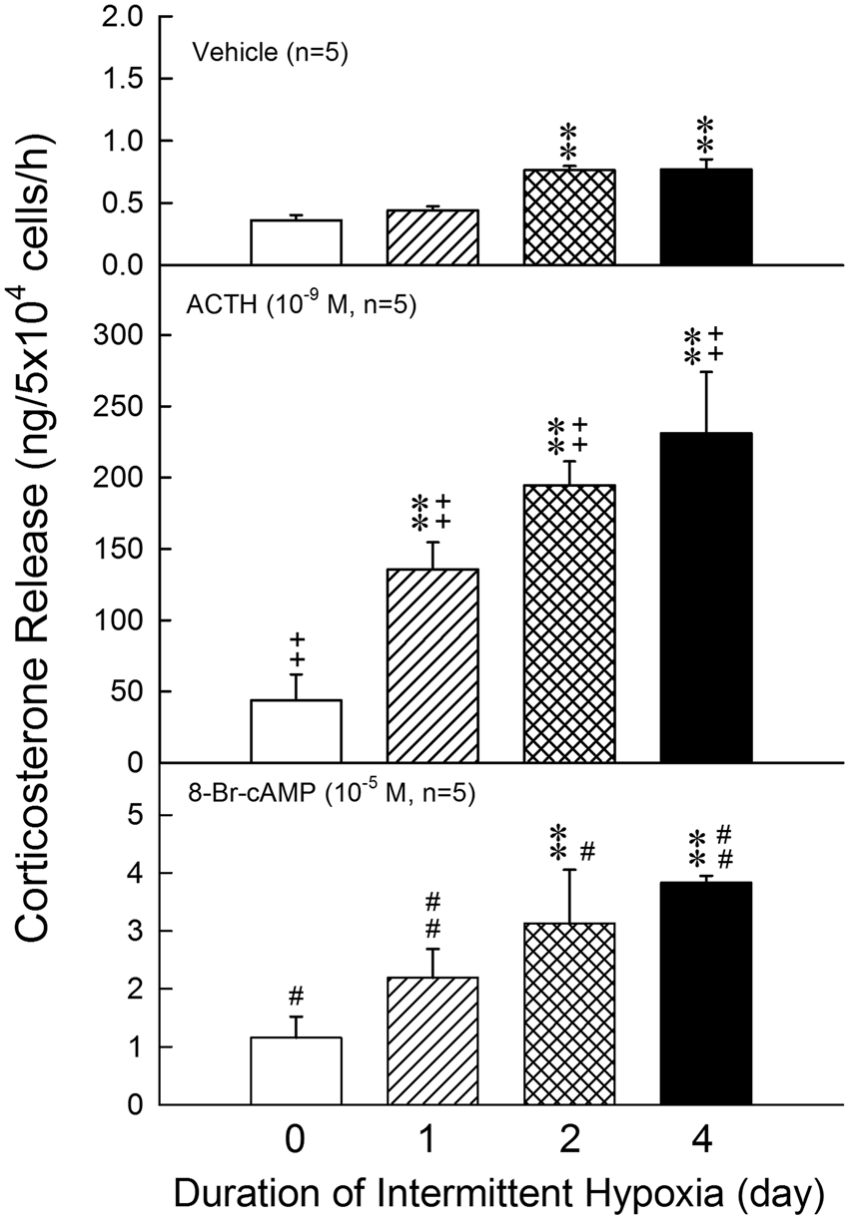
Effects of hypoxia on the basal, ACTH- or 8-Br-cAMP-stimulated corticosterone release in ZFR cells. **P < 0.01 compared to normoxia (control) group. ^++^ *P* < 0.01 compared to control group. ^#^ *P* < 0.05, ^##^ *P* < 0.01 compared to control group by Student’s *t*-test. Each value represents the mean ± SEM.

After intermittent hypoxia for 2 or 4 days, the basal release of corticosterone by ZFR cell was markedly increased (*P* < 0.01) (Fig. 2, top panel). Incubation of ACTH (10^−9^ M) produced significant (*P* < 0.01) increase in corticosterone release by ZFR cells in both normoxia [F = 7.86, df = 2, *P* = 0.004] and intermittent hypoxia [Day 1: F = 76.74, df = 2, *P* < 0.001; Day 2: F = 158.22, df = 2, *P* < 0.001; Day 4: F = 223.17, df = 2, *P* < 0.001;] groups (Fig. 2, central panel). The corticosterone release in response to ACTH was further enhanced in intermittent hypoxia [F = 85.56, df = 3, *P* < 0.001] as compared with normoxia group (Fig. 2, central panel). Application of 8-Br-cAMP significantly elevated the corticosterone release by ZFR cells in all groups (Student’s *t*-test: *P* < 0.05, *P* < 0.01) (Fig. 2, bottom panel). As compared with normoxia group, a further increase of corticosterone stimulated by 8-Br-cAMP was observed in 2- and 4-day intermittent hypoxia groups (Fig. 2, bottom panel).

### Effects of intermittent hypoxia on the activity of calcium channel associated corticosterone release in rat ZFR cells

**Figure 3:**
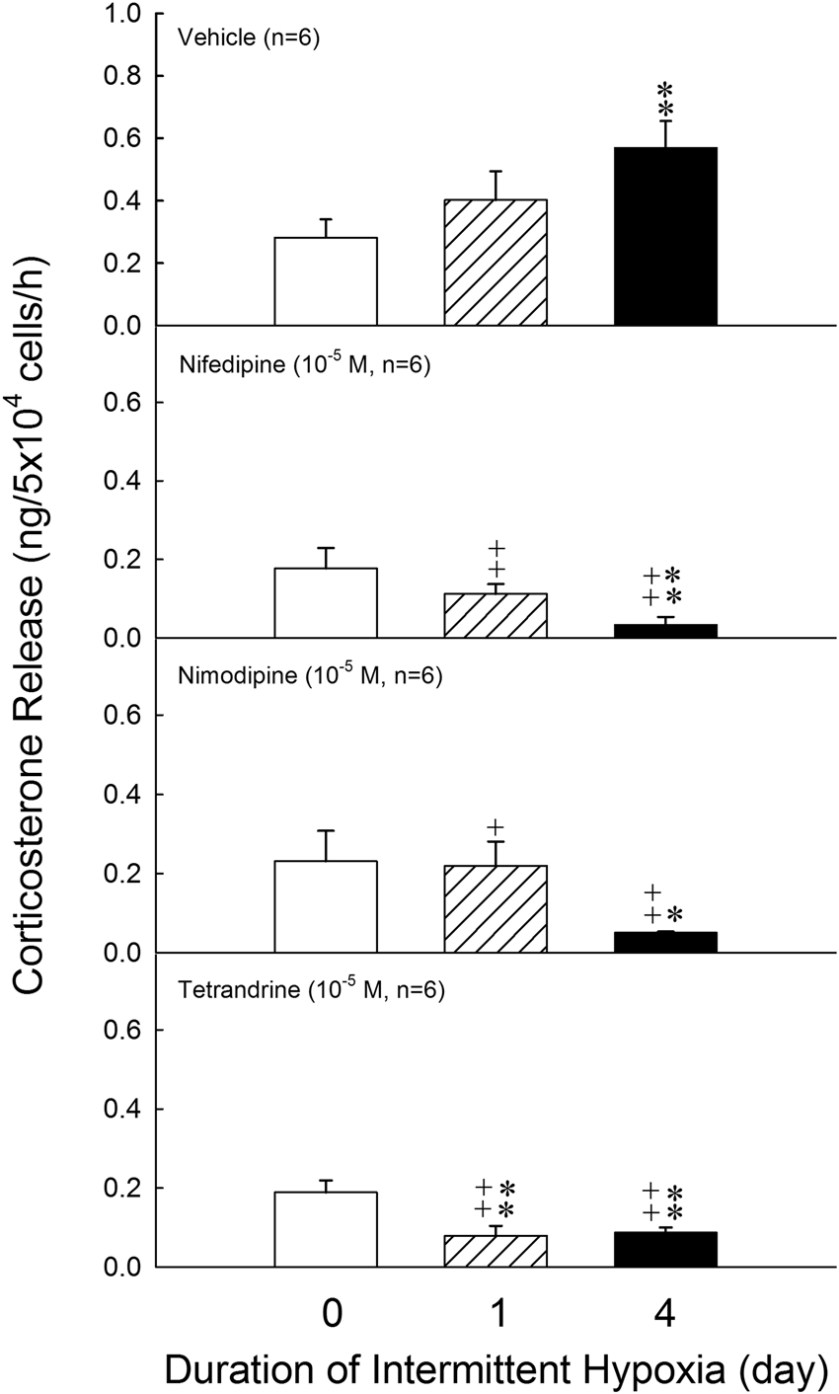
Effects of hypoxia on the activity of calcium channel associated corticosterone release in ZFR cells. *P < 0.05, **P < 0.01 compared to normoxia (control) group. ^**+**^ *P* < 0.05, ^++^ *P* < 0.01 compared to calcium blocker. Each value represents the mean ± SEM.

The involvement of calcium channels in regulation of intermittent hypoxia-related corticosterone release was examined. The variant calcium channel blockers including nifedipine (an L-type calcium blocker), nimodipine (an L-type calcium blocker) and tetrandrine (T-type calcium blocker) were employed. The results were illustrated in Fig. 3. In the top panel, the corticosterone release was higher in 4-day intermittent hypoxia than in normoxia group [F = 21.80, df = 2, *P* = 0.008]. Nifedipine, nimodipine and tetrandrine did not alter the corticosterone release in normoxia group. The elevated corticosterone release after intermittent hypoxia for 1 day and 4 days were attenuated [Day 1: F = 22.23, df = 3, *P* = 0.001, 0.012, 0.001; Day 4: F = 69.39, df = 3, *P* < 0.001] by treatment with nifedipine, nimodipine and tetrandrine. The corticosterone release in ZFR cells of 4- day intermittent hypoxia group was decreased by the administration of tetrandrine [F = 3.95, df = 2, *P* = 0.003], nimodipine [F = 10.81, df = 2, *P* = 0.014] and nifedipine [F = 5.46, df = 2, *P* = 0.004] (Fig. 3).

### Effects of intermittent hypoxia on the activity of P450scc and 3β-HSD in ZFR cells

**Figure 4:**
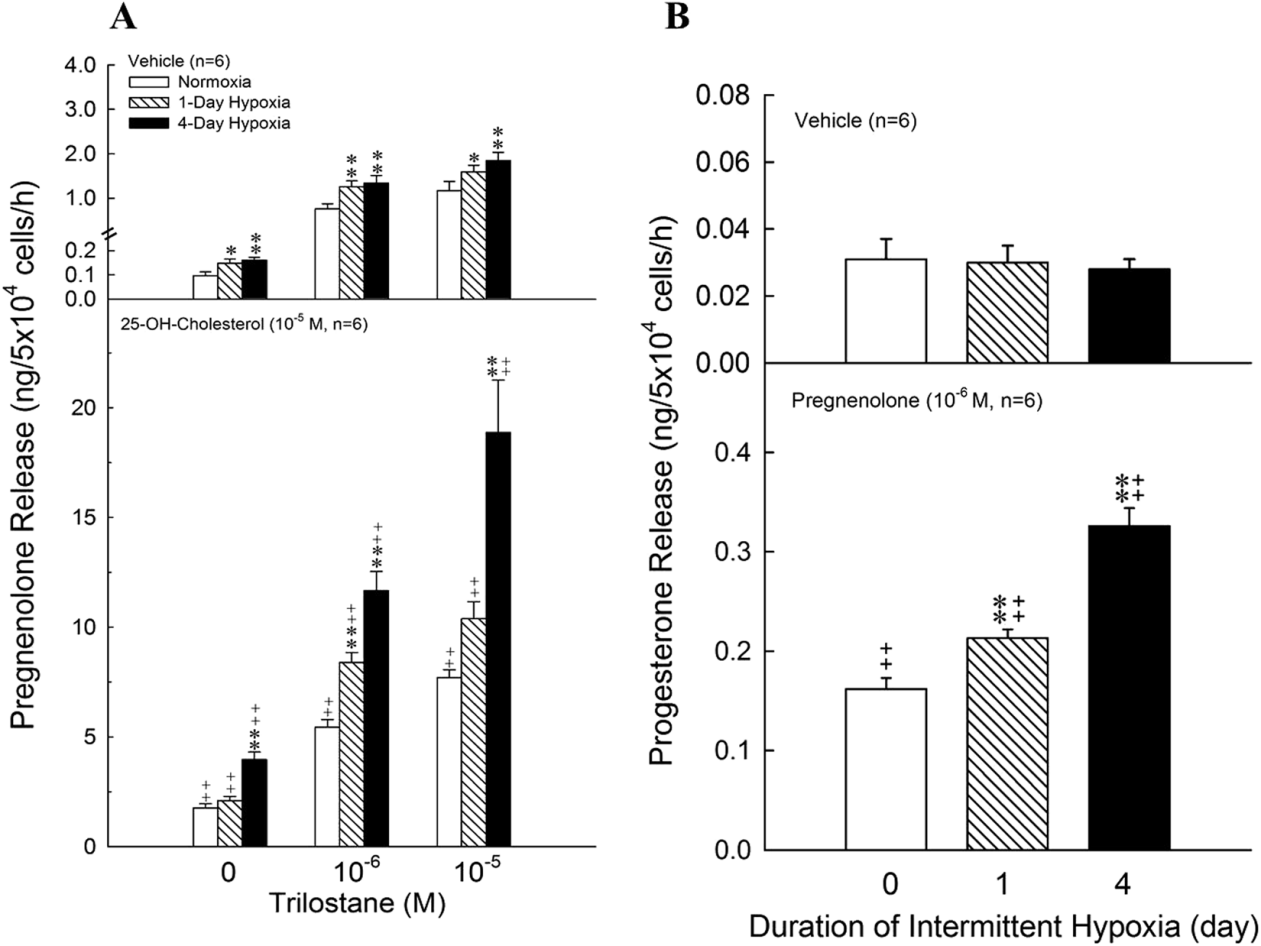
Effects of hypoxia on the activity of P450scc and 11β-HSD in ZFR cells. (**A**) *P < 0.05, **P < 0.01 compared to normoxia (control) group. ^++^ *P* < 0.01 compared to trilostane = 0, 10^−6^ or‘ 10^−5^ M. Each value represents the mean ± SEM. (**B**) **P < 0.01 compared to normoxia (control) group. ^++^ *P* < 0.01 compared to control group. Each value represents the mean ± SEM.

The results of the conversion of 25-OH-cholesterol (10^−5^ M) to pregnenolone in the presence of trilostane (10^−6^ and 10^−5^ M) in the ZFR cells of normoxia and intermittent hypoxia rats were illustrated in Fig. 4A. Incubation of ZFR cells with 25-OH-cholesterol (10^−5^ M) markedly increased the pregnenolone production in all groups [F = 131.18-2260.62, df = 1, *P* = 0.000011–0.000106] (Fig. 4A). Treatment of trilostane alone resulted in greater pregnenolone production in 4-day intermittent hypoxia groups than in normoxia group [Trilostane = 10^−6^ M: F = 150.94, df = 2, *P* < 0.0001; Trilostane = 10^−5^ M: F = 94.03, df = 2, *P* < 0.0001] (Fig. 4A). The combination of 25-OH-cholesterol and trilostane resulted in a more accumulation of pregnonolone as compared with the group of trilostane or 25-OH-cholesterol alone in normoxic and intermittent hypoxic groups. Compared with the normoxia group, 25-OH-cholersterol plus trilostane caused higher pregenenolone production (i.e. high activity of P450scc) in 4-day intermittent hypoxia groups (Fig. 4A, *P* < 0.01).

The results of conversion of pregnenolone to progesterone in normoxic and intermittent hypoxic groups were shown in Fig. 4B. Treatment of pregnenolone markedly increased the progesterone production in all groups [F = 212.75, df = 2, *P* = 0.006, 0.000]. The production of progesterone was significantly higher in 1-day and 4-day intermittent hypoxia groups than in normoxia group [Day 1: F = 505.71, df = 1, *P* < 0.000007; Day 4: F = 1341.00, df = 1, *P* < 0.000009].

### Effects of intermittent hypoxia on the enzyme activities during steroidogenesis of corticosterone in ZFR cells

**Figure 5:**
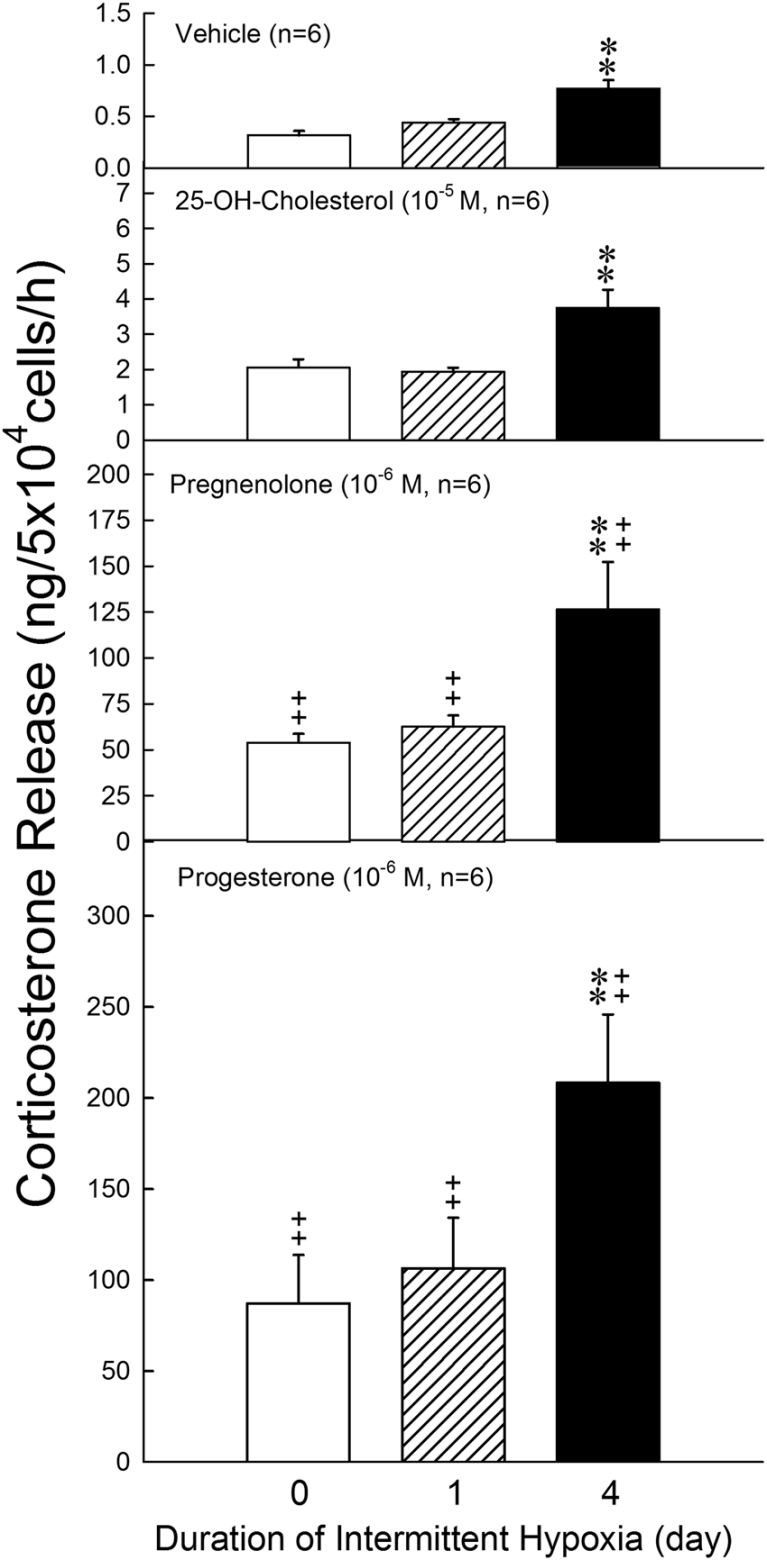
Effects of hypoxia on the steroidogenesis of corticosterone in reponse to 3 parameters in ZFR cells. **P < 0.01 compared to normoxia (control) group. ^++^ *P* < 0.01 compared to control group. Each value represents the mean ± SEM.

The increased level of corticosterone secretion stimulated by 25-OH-cholesterol was greater in the 4-day intermittent hypoxia group than in the normoxia or 1-day intermittent hypoxia group [F = 0.01, df = 2, *P* = 0.001] (Fig. 5, second panel). The precursors for biosynthesis of corticosterone including pregnenolone, progesterone, and deoxycorticosterone were also employed to determine if intermittent hypoxia changes the enzyme activities of steroidogenesis. Administration of pregnenolone or progesterone significantly elevated the corticosterone release in normoxia [F = 17.85, df = 3, *P* = 0.003, *P* < 0.001] and intermittent hypoxia groups [Day 1: F = 26.32, df = 3, *P* = 0.002, *P* = 0.000; Day 4: F = 101.96, df = 3, *P* < 0.001, *P* < 0.001]. After intermittent hypoxia treatment for 4 days, the corticosterone release stimulated by precursors (pregnenolone and progesterone) was greater than those in normoxia and 1-day intermittent hypoxia groups (Fig. 5).

### Effects of intermittent hypoxia on the activity of 11β-hydroxylase in ZFR cells

**Figure 6:**
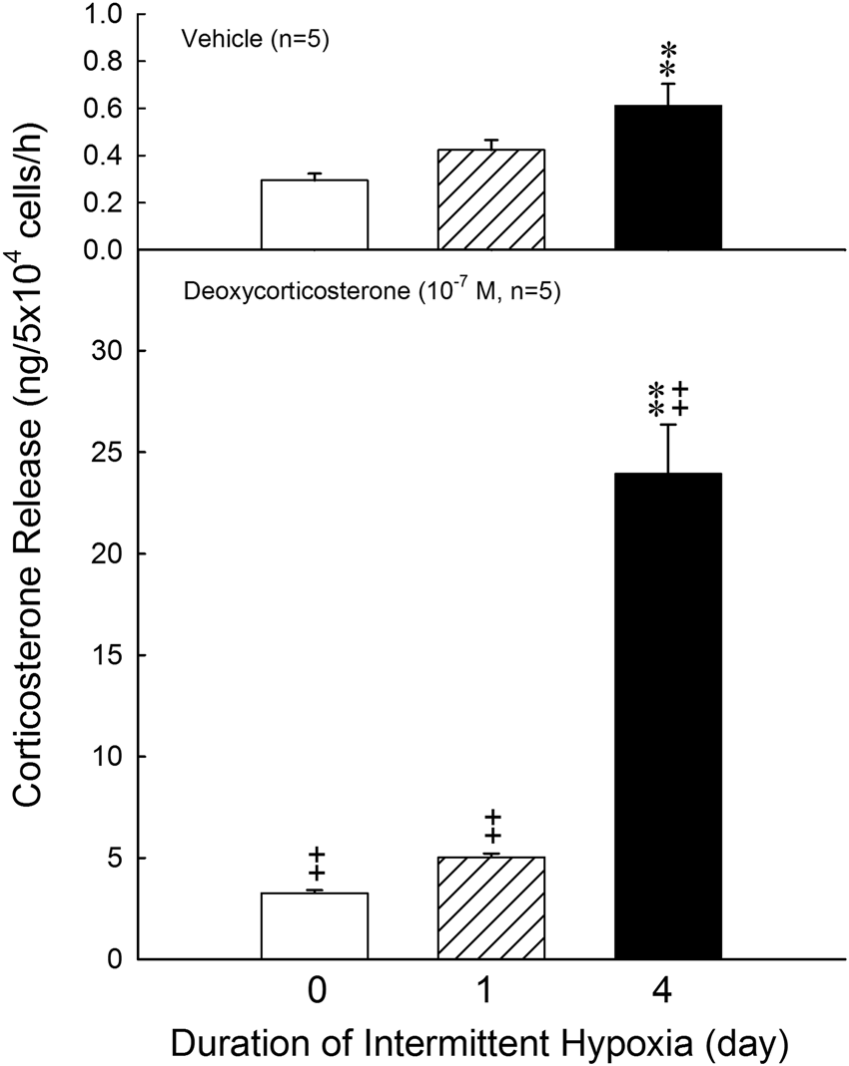
Effects of hypoxia on the activity of 11β-hydroxylase in ZFR cells. **P < 0.01 compared to normoxia (control) group. Each value represents the mean ± SEM. ^++^ *P* < 0.01 compared to deoxycorticosterone. Each value represents the mean ± SEM.

Figure 6 showed the results of conversion of deoxycorticosterone to corticosterone in the normoxic and intermittent hypoxic groups. Application of deoxycorticosterone significantly elevated the corticosterone secretion in the normoxic [F = 13.25, df = 1, *P* = 0.000004] and intermittent hypoxic groups [Day 1: F = 31.97, df = 1, *P* = 0.000002; Day 4: F = 816.85, df = 1, *P* = 0.000077]. The release of corticosterone in reponse to deoxycorticosterone was further enhanced by 4-day intermittent hypoxia as compared with normoxia group [F = 394.14, df = 2, *P* < 0.001].

### Effects of intermittent hypoxia on protein expressions of ACTH-R, StAR and P450scc

**Figure 7:**
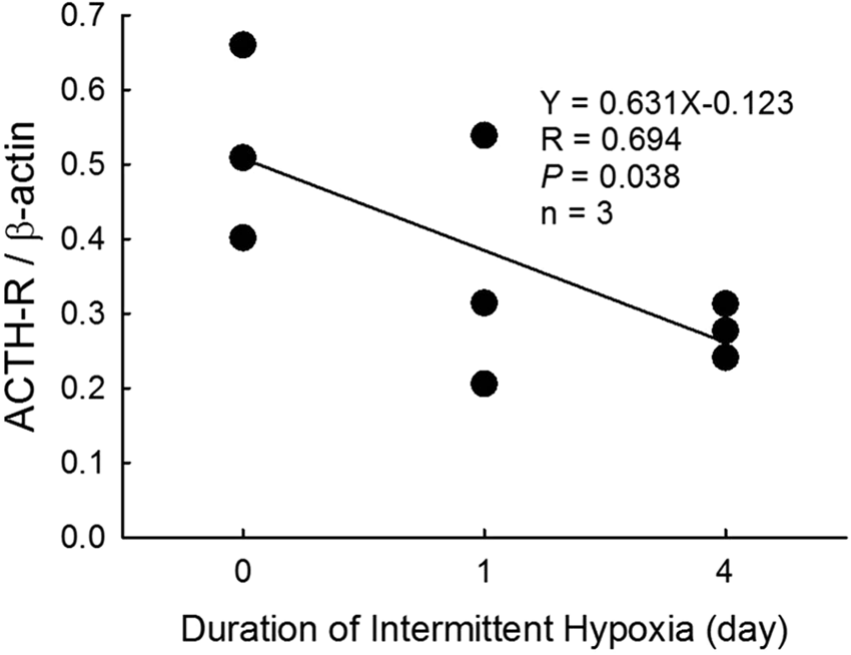
The correlation analysis between hypoxic days and protein expression of ACTH receptor. Each value represents the mean ± SEM. The protein expressions of ACTH-R were significantly down-regulated followed by the hypoxic duration (*P* = 0.038, R = 0.694).

**Figure 8:**
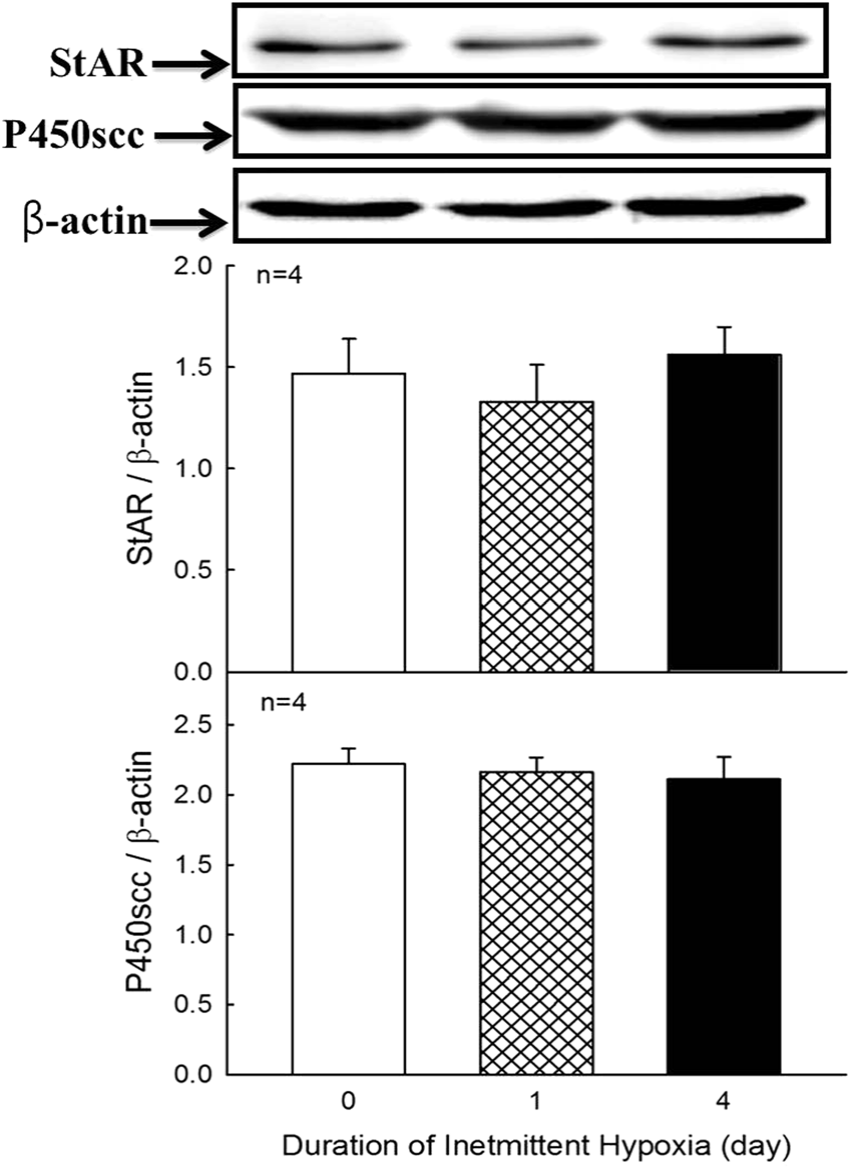
Effects of hypoxia on the protein expression of StAR and P450scc in ZFR cells. Each value represents the mean ± SEM. Treatment of intermittent hypoxia for 1 or 4 days did not alter the protein expression of either the StAR or P450scc in rat ZFR cells.

The protein expressions of ACTH-R were decreased by treating of intermittent hypoxia for 1 or 4 days in the ZFR cells as compared with normoxic group (Fig. 7, *P* < 0.05, R = 0.694). Moreover, the protein expression of neither StAR nor P450scc was altered by intermittent hypoxia (Fig. 8).

## Discussion

The present results suggested that intermittent hypoxia increased the production of corticosterone in rats, via an increase of the activities of [1] c-AMP [2] L- and T-type calcium channels as well as enzyme activities of P450scc, 3β-HSD, 21β-hydroxylase and 11β-hydroxylase.

Adrenal steroid biosynthesis responds to stimulation with ACTH secreted from the anterior pituitary gland. In adrenal cortical tissues, cytochrome P450s (P450scc, P450c21, P450c17, P450c11, etc.) and hydroxysteroid dehydrogenases (3β-HSD, 21β-HSD and 11β-HSD) are utilized as catalysts of the biosynthesis pathway^25^. These enzymes catalyze a cascade of reactions converting cholesterol ultimately into final steroid products. Despite differences in the expression profiles of steroidogenic enzymes, steroidogenesis in each cell type is stimulated by the same cAMP/PKA-signaling pathway^26, 27^.

On the other hand, the secretion of corticosterone was affected by cellular Ca^2+^ concentration. Administration of cilnidipine (L- and N-type dual calcium channel blocker) and nimodipine (an L-type calcium channel blocker) significantly attenuated the immobilized stress-induced behavioral changes and restored memory deficits along with normalization of the corticosterone level^28^. It has been suggested that cilnidipine and nimodipine attenuate corticosterone release by blockage of calcium channels (both L- and N-type) on the HPA-axis, which is responsible for beneficial effects in restoration of behavioral alterations and memory deficits in immobilization induced acute stress in mice.

The present studies demonstrated that administration of L-type calcium channel blocker (nifedipine or nimodipine, 10^−5^ M) and T-type calcium channel blocker (tetrandrine, 10^−5^ M) did not alter the release of corticosterone from ZFR cells, but the elevated corticosterone release after intermittent hypoxia was attenuated by treatment with the above calcium channel blockers. These results suggested that intermittent hypoxia potentiated the calcium channel activities in response to the above blockers. In another words, the activations of both L-type and T-type calcium channels are involved in the action mechanism of intermittent hypoxia on corticosterone release from ZFR cells.

In our previous studies, we found that the intermittent hypoxia stimulated the secretion of testosterone via stimulatory actions on the activities of adenylyl cyclase, cAMP, L-type calcium channel, and steroidogenic enzyme^29^. Meanwhile, the progesterone production decreased with decreasing O_2_ concentration in luteal cells, although which was significantly stimulated by LH regardless of O_2_ concentration. Low-oxygen condition also inhibited pregnenolone production and P450scc mRNA expression was decreased in both non-LH-treated and LH-treated status^30^. Hypoxia also acts on the hypothalamus-pituitary-adrenal axis to increase the corticotroph number and the levels of plasma ACTH^31^. An increase of the concentration of corticosterone was demonstrated following 2 days of hypobaric hypoxia^32^. Hypoxia-inducible factor 1 alpha (HIF1α) appears to be a positive regulator of basal and stimulated steroid acute regulatory protein (StAR) expression, which under partial hypoxia is capable of increasing the steroidogenic capacity of granulosa cells^33^. The hypoxic microenvironment imposes a metabolic adaptation to macrophages, skewing their functions towards a mitogenic, pro-invasive, pro-angiogenic and pro-metastatic phenotype, thus supporting tumor growth^34, 35^. The corticosteroid affects a wide range of physiological processes, including glycogenesis, hepatic gluconeogenesis and protein catabolism, as well as immunosuppressive and hypertensive effects^36–39^.

It has been shown that hypoxia activates the secretion of ACTH via cAMP generation and chronic hypoxia can inhibit the function of adrenocotical cells of rats^40^. Hypoxia significantly increased corticosterone production without affecting ACTH secretion^41^. Transitioning fetal adrenal cells from hypoxia to normoxia increased mRNAs for P450c17, 3βHSD2, and StAR^42^. It has been demonstrated that rats with exposure to 5 or 7 km altitude of hypoxia for a short or long term can significantly enhance CRH release in the median eminence and resulted in stimulating corticosterone secretion^43^. Besides, a significant increase in plasma corticosterone was observed in chronic intermittent hypoxia rats compared to the control^44^. In the fetuses (134–136 days), hypoxemia resulted in significantly increased levels of mRNAs encoding P450scc, 21β-hydroxysteroid dehydrogenase and 3β-hydroxysteroid dehydrogenase. These results suggested that hypoxemia is a potent stimulant to activate adrenal steroidogenesis in fetal sheep during late gestation^32^. Rat pups exposed to hypoxia from birth display ACTH-independent increases in corticosterone by increasing StAR, P450scc and peripheral-type benzodiazepine receptor (PBR) proteins. These results indicated ACTH-independent increase in corticosterone might be the reason for the pups to increase circulating glucocorticoids for survival^45^.

**Figure 9:**
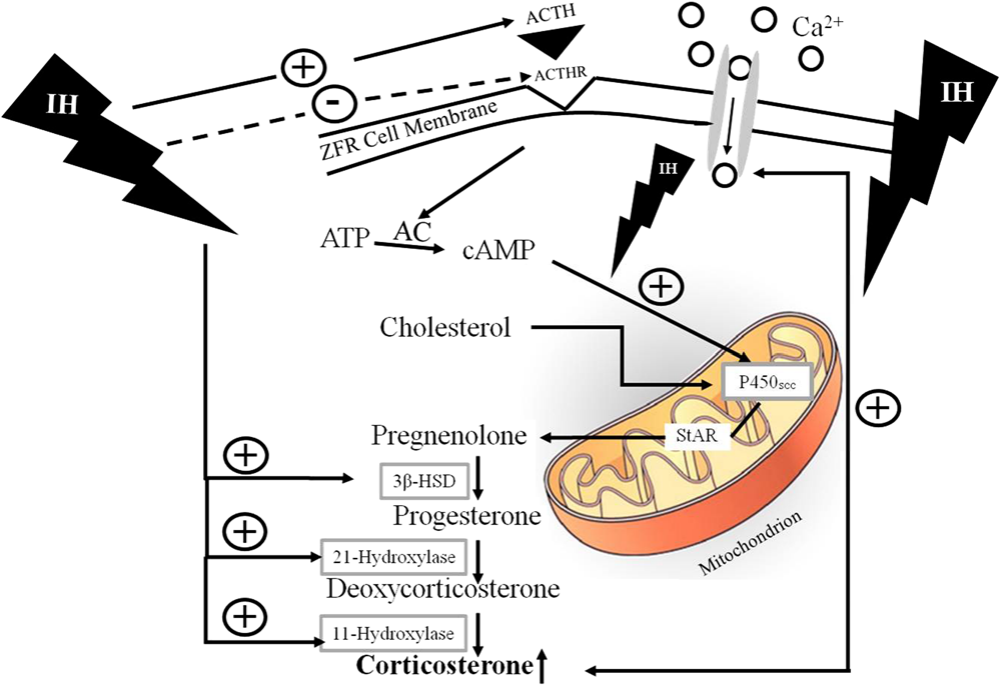
Schmatic representation of the stimulation of the biosynthesis and recreation of corticosterone by intermittent hypoxia in rat ZFR cells. IH, intermittent hypoxia; ACTH, adrenocorticotropin; StAR, steroidogenic acute regulatory protein; 3β-HSD, 3β-hydroxysteroid dehydrogenase; ZFR cells, zona-fasciculata-reticularis cells. The solid line represents stimulation. The dash line represents inhibition.

Our studies were designed to explore the mechanisms of the corticosterone production in ZFR cells after intermittent hypoxia *in vivo*. The present results indicated that basal corticosterone increased after intermittent hypoxia. Corticosterone increased significantly by intermittent hypoxia following ACTH or c-AMP treatment. After tetrandrine (T-type calcium blocker) challenge alone, the corticosterone concentration was inhibited by 1-day hypoxia. Administration of nifedipine (an L-type calcium blocker), nimodipine (an L-type calcium blocker) or tetrandrine resulted in a deep reduction of corticosterone secretion by 4-day intermittent hypoxia as compared with normoxic group. We therefore suggested that the enhanced secretion of corticosterone was at least in part via a calcium-signaling messenger mechanism after intermittent hypoxia. Treatment of trilostane or 25-OH-cholesterol alone resulted in a greater pregnenolone production after intermittent hypoxia. These results suggested that enzyme activities for corticosterone biosynthesis including P450scc, 3β-HSD, and 21β-hydroxylase, in ZFR cells were upregulated by 2–3 folds after intermittent hypoxia treatment. Treatment with deoxycorticosterone resulted in a 5-fold increase of corticosterone production in hypoxia, which suggested that 11β-hydroxylase activity was upregulated after intermittent hypoxia treatment *in vitro*. We confirmed the activities of P450scc, 3β-HSD, and 21β-hydrpxylase were upregulated after intermittent hypoxia and the steroids of these enzymes were accumulated or accelerated through these products to become deoxycorticosterone and made deoxycorticosterone accumulation. Then the activity of 11β-hydroxylase was enhanced and corticosterone production was upregulated by 5 folds. Since the protein expressions of P450scc and StAR were not altered by the administration of intermittent hypoxia (Fig. 8), the increased production of corticosterone in response to intermittent hypoxia in rat ZFR cells is independent of the generation of P450scc and StAR. Although the increased secretion of ACTH in response to hypoxia had been observed^32, 33^, the increased co rticosterone production without affecting ACTH secretion after hypoxia administration was also reported. In the present study, we found that the protein expression of ACTH-R in rat ZFR cells was down-regulated by the administration of intermittent hypoxia (Fig. 7). This might be due to the negative feedback of increased corticosterone on the expressions of ACTH-R to regulate the release of ACTH. The findings and observations of the present study have been illustrated in Fig. 9.

In these studies, we concluded that intermittent hypoxia increased the secretion of corticosterone of rats was at least via 3 effects: [1] to increase intracellular actions and functions of ACTH and cAMP, [2] to act on T- and L-type calcium channels to enhance the secretion of corticosterone, and [3] to increase the activities of steroidogenic enzymes including P450scc, 3β-HSD, 21β-hydroxylase and 11β-hydroxylase to enhance the release of corticosterone.
